# The Autoantibody Array Assay: A Novel Autoantibody Detection Method

**DOI:** 10.3390/diagnostics13182929

**Published:** 2023-09-13

**Authors:** Yuta Norimatsu, Kazuki Mitsuru Matsuda, Kei Yamaguchi, Chihiro Ono, Taishi Okumura, Emi Kogo, Hirohito Kotani, Teruyoshi Hisamoto, Ai Kuzumi, Takemichi Fukasawa, Asako Yoshizaki-Ogawa, Naoki Goshima, Shinichi Sato, Ayumi Yoshizaki

**Affiliations:** 1 Department of Dermatology, The University of Tokyo Graduate School of Medicine, Tokyo 113-8655, Japan; norimanorima@gmail.com (Y.N.); takemichi.giraffe@gmail.com (T.F.); asako56planetes730@yahoo.co.jp (A.Y.-O.);; 2 Department of Dermatology, International University of Health and Welfare Narita Hospital, Chiba 286-8520, Japan; 3 Molecular Profiling Research Center for Drug Discovery, National Institute of Advanced Industrial Science and Technology, Tokyo 100-0013, Japan; 4 ProteoBridge Corporation, Tokyo 135-0064, Japan; 5 Department of Clinical Cannabinoid Research, The University of Tokyo Graduate School of Medicine, Tokyo 113-8655, Japan

**Keywords:** systemic sclerosis, dermatomyositis, cell-free protein synthesis, autoimmune collagen diseases

## Abstract

Systemic sclerosis (SSc) and dermatomyositis (DM) are autoimmune collagen diseases. Specific autoantibodies are known to be involved in their pathogeneses, each presenting with a different clinical manifestation. Although immunoprecipitation is the gold standard method for detecting autoantibodies, it is difficult to perform in all cases owing to the use of radioisotopes. In this study, we developed a new detection method for SSc and DM autoantibodies (A-cube) using cell-free protein synthesis and examined its validity. Proteins were synthesized using wheat germ cell-free protein synthesis. A total of 100 cases of SSc, 50 cases of DM, and 82 healthy controls were examined. The validity of the method was examined by a comparison with existing test results. Anti-centromere antibody, anti-topoisomerase I antibody, anti-RNA polymerase III antibody, anti-U1RNP anti-body, anti-Jo-1 antibody, anti-TIF1γ antibody, anti-Mi-2 antibody, and anti-ARS antibody were tested for. The results suggested that A-cube is comparable with existing testing methods or has a high sensitivity or specificity. In addition, there was a case in which the diagnosis was reconsidered using the A-cube. The quality of the A-cube was ensured, and its usefulness for a comprehensive analysis was demonstrated. The A-cube can therefore contribute to the clinical assessment and treatment of SSc and DM.

## 1. Introduction

Systemic sclerosis (SSc) is a collagen disease that involves three pathological conditions: autoimmunity, vascular damage, and fibrosis [1]. B cells are thought to play an important role in the pathogenesis of SSc, but recently, Th17 has also been thought to play an important role and is attracting attention as a new therapeutic target for SSc [2,3,4]. Autoantibodies such as anti-topoisomerase I, anti-centromere, and anti-RNA polymerase III (RNAP3) are known to be involved in the pathogenesis of SSc, and each has a different clinical appearance. For example, anti-centromere antibody-positive systemic sclerosis often results in localized systemic sclerosis, whereas anti-topoisomerase I antibody-positive systemic sclerosis and anti-RNAP3 antibody-positive systemic sclerosis result in diffuse systemic sclerosis. In anti-topoisomerase I-positive systemic sclerosis, interstitial pneumonia should be considered, whereas in anti-RNAP3 antibody-positive systemic sclerosis, severe skin sclerosis and complications of malignancy should be considered [5]. These autoantibodies are also known to appear prior to the characteristic symptoms of SSc, and their detection is considered to be important in the diagnosis of early and mild cases [6]. In addition, it is believed that the autoantibodies characteristic of SSc do not change to other antibodies or disappear spontaneously once they have appeared, and the autoantibody titer of the anti-topoisomerase I antibody is correlated with the modified Rodnan skin score (mRSS), the presence of ulcers, and the severity of interstitial pneumonia [7,8,9]. Thus, the measurement of the autoantibody titer characteristic of SSc is considered to be important not only for diagnosis, but also for understanding the disease status.

Dermatomyositis (DM) is also an autoimmune disease, in which characteristic autoantibodies such as anti-MDA5, anti-Jo-1, and anti-TIF1γ are involved in its pathogenesis, each presenting a different clinical picture [10,11,12]. 

Immunoprecipitation is the gold standard for detecting autoantibodies [13]. However, because immunoprecipitation uses radioisotopes, it is difficult to perform immunoprecipitation in all cases; therefore, analyses are often limited to immunoblotting and ELISA [13]. Although immunoblotting is a simpler method than immunoprecipitation, it is not a useful test because of its high false negative rate of 19% [14,15]. In particular, the false negative rate of immunoblotting for anti-OJ antibodies is 100%, and it is thought that anti-OJ antibodies cannot be detected by immunoblotting.

It is also known that isoleucyl-tRNA synthetase (IARS), which is considered to be a major anti-OJ antibody antigen, does not react with anti-OJ antibodies, even if it is prepared via the existing ELISA method using *Escherichia coli* [16]. Major anti-OJ antibody antigens are glutamyl-prolyl-tRNA synthetase (EPRS), leucine tRNA synthetases, methotionyl-tRNA synthetase (MARS), glutaminyl-tRNA synthetase (QARS), lysyl-tRNA synthetase (KARS), and arginyl-tRNA synthetase (RARS). Anti-OJ antibody antigen is a component of the enzyme complex consisting of eight aminoacyl-tRNA synthetases (EPRS, MARS, QARS, KARS, RARS, and aspartyl-tRNA synthetase (DARS)), along with the aminoacyl- tRNA synthetase complex interacting with (AIMP)1, AIMP2, and AIMP3. It has been suggested that the higher-order structure of the enzyme complex may be important for the recognition of anti-OJ antibodies [16,17]. Thus, the detection of autoantibodies is difficult in some cases using the existing test methods. 

The wheat germ cell-free system is known to have higher expressions of proteins of any molecular weight than existing protein synthesis systems, such as *E. coli* and silkworm. Conventional tests have used *E. coli* to synthesize proteins, but the number of proteins that can be synthesized is limited, because they are prokaryotic organisms and their translation reaction patterns are different from those of humans. The seed embryos of higher organisms, such as wheat germ, are thought to be excellent materials for the preparation of cell-free systems because they store a large amount of highly active translation factors (50% of the germ weight) in preparation for germination. On the other hand, it is known that the method using these higher organisms is, at the same time, susceptible to translation inhibitory factors, and protein synthesis tends to be unstable. The germ cell-free system was thought to be as unstable as other synthesis methods because of the translation enzyme system, however, the reaction duration and amount of protein synthesized have been improved by washing, and the germ cell-free system is now attracting attention as a useful method [18,19,20]. Therefore, in this study, we developed a new test method known as A-cube using this technology and validated it by comparing it with the existing test methods. Our results suggest that A-cube is comparable with existing tests or has a high sensitivity or specificity. Additionally, A-cube detected anti-OJ antibodies that could not be detected by conventional tests. 

## 2. Materials and Methods

### 2.1. Patients

We studied 100 patients diagnosed with SSc, 50 patients with DM, and 82 healthy participants at our hospital. SSc was diagnosed using the 2013 American College of Rheumatology (ACR) criteria [21]. DM was diagnosed using the 2017 EULAR/ACR criteria [22]. The sera of patients with benign skin tumors who visited the dermatology department of our hospital and provided consent were used as controls. The backgrounds of the patients with SSc and DM and the healthy participants are shown in Table 1. We excluded serum when the patient was suffering from an infectious disease.

### 2.2. Specimens

Sera stored at −80 °C in our hospital were used. 

### 2.3. Preparation of New Arrays

**Figure 1:**
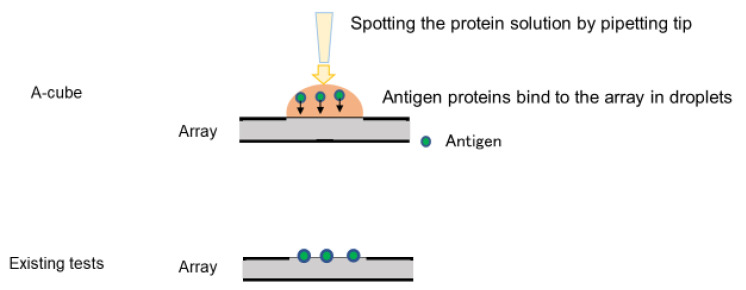
Characteristics of A-cube.

Wheat germ cell-free protein synthesis technology was selected as the protein synthesis system [18,19,20]. The synthesized proteins were captured on array plates under wet conditions. For preparing the array plates, amino-group-modified glass plates (SDM0011, Matsunami Glass, Osaka, Japan) were coated with 50 mM of glutathione (GSH) via Sulfo-SMPB (22317, Thermo Fisher Scientific, Waltham, MA, USA). The translation reaction mixture containing the FLAG-GST-tagged target protein was diluted 5 times with PBS and simultaneously spotted onto 4 GSH-coated glass plates (240 spots/plate) using a 1536-channel independent cylinder system (BIOTEC, Tokyo, Japan). The translation reaction mixture was spotted in duplicate. After spotting, the plates were incubated at room temperature for 30 min and washed with Tris-buffered saline containing 0.1% Tween 20 (TBST; 9997S, Cell Signaling Technology, Danvers, MA, USA). The plates were then incubated in blocking buffer (50 mM HEPES pH 7.5, 200 mM NaCl, 0.08% Triton-X, 25% Glycerol, 5 mM GSH, 0.3% skim milk, and 1 mM DTT) and stored at −80 °C until use. The autoantibody assay using this array plate was named A-Cube. To maintain the higher-order structure of the synthesized proteins, a solution containing the undenatured antigen proteins was spotted on the substrate and frozen (Figure 1), which enabled us to keep the proteins in a wet state without drying until the serum reaction took place.

Unlike existing testing, the A-cube did not dry once before measurement because the protein solution was spotted onto the array as it was.

The existing tests used for comparison included:

- Anti-centromere antibody (MESACUP-2)
- Anti-Topo1 antibody (MESACUP-3)
- Anti-RNAP3 antibody (IMESACUP)
- Anti-U1RNP antibody (ThermoFisher)
- Anti-Jo-1 antibody (MESACUP-3)
- Anti-TIF1γ antibody (MESACUP)
- Anti-Mi-2 antibody (MESACUP)
- Anti-ARS antibody

A statistical analysis was performed using Spearman’s rank correlation test, with statistical significance defined as *p* < 0.05. Prism 8 (GraphPad Software, San Diego, CA, USA) was used for all the statistical analyses.

## 3. Results

### 3.1. Determination of Reference Values

The reference values were determined using the measurement results of the 82 healthy participants. The reference values were determined based on the mean +3 SD and mean +5 SD. CENP-A, RNAPII, and SMN were classified as follows: (Unit_Index) (−): less than 10.0; (±): greater than 10.0, to less than 13.0; and (+): greater than 13.0. Other antibodies were classified as (Unit_Index) (−): less than 7.0; (±): greater than 7.0, to less than 10.0; and (+): greater than 10.0.

### 3.2. For Healthy Participants

It is known that various antibodies, such as antinuclear antibodies, are positive in healthy participants [23]; thus, the fact that 100% specificity was not achieved for all antibodies was considered to be a natural result.

### 3.3. Anti-Centromere Antibodies

Of the 20 ELISA (CENP-B)-positive cases, all 20 were consistent with the A-cube-positive cases (Table 2). One case was negative for ELISA and positive for A-cube (Table 2). This case was followed-up for limited cutaneous SSc (lcSSc) with an unknown antibody. Additional antinuclear antibodies were submitted by our department, and the result was centromere pattern positive. This suggested that this was a false negative ELISA case. Therefore, anti-centromere antibodies are considered to be more sensitive to the A-cube than MESACUP. 

Table 2 shows the results for each antibody.

**Figure 2:**
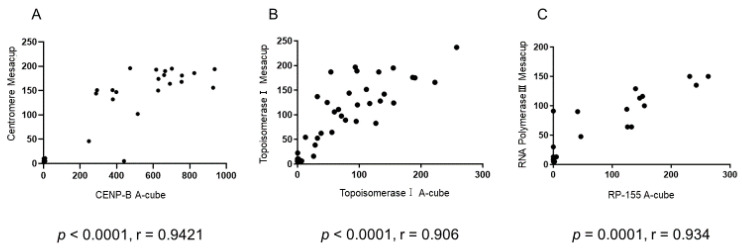
Comparison of autoantibody titers measured by A-cube with autoantibody titers measured by the existing test. (**A**) Autoantibody titers of anti-centromere antibodies measured with A-cube correlated with autoantibody titers measured with MESACUP, an existing test method (*p* < 0.0001, r = 0.9421). (**B**) Autoantibody titers of anti-topoisomerase I antibodies measured by A-cube correlated with those measured by MESACUP, an existing test method (*p* < 0.0001, r = 0.906). (**C**) Autoantibody titer of anti-RNA polymerase III antibody measured by A-cube correlated with autoantibody titer measured by MESACUP, an existing test method (*p* = 0.0001, r = 0.934).

The correlation between the autoantibody titer of the existing test (MESACUP-2) and the autoantibody titer detected by the A-cube was examined, and the result was significant (*p* < 0.0001, (r = 0.9421)), indicating a good correlation (Figure 2A). 

### 3.4. Anti-Topoisomerase I Antibody

Of the 31 ELISA (topoisomerase I)-positive cases, 30 were positive for the A-cube (Table 2). In one case, the antibody titer was weakly positive (22.5) using ELISA and no anti-topoisomerase I antibody was detected using immunoprecipitation, suggesting a false positive ELISA. Of the 67 ELISA-negative cases, all 67 were consistent with the A-cube-negative cases (Table 2).

Therefore, the anti-topoisomerase I antibody is considered to be more specific to the A-cube than MESACUP3. 

In addition, the correlation between the autoantibody titer of the existing test (MESACUP-3) and the autoantibody titer detected by the A-cube was examined, and the result was significant (*p* < 0.0001, (r = 0.906)), indicating a good correlation (Figure 2B).

### 3.5. Anti-RNA Polymerase III Antibody

Of the 14 ELISA (RP155)-positive cases, 13 were A-cube-positive (Table 2). This case was considered a false positive case because the antinuclear antibody was also negative and the ELISA titer was low. Of the 75 ELISA-negative cases, all 75 were A-cube-negative (Table 2).

Therefore, RP155 was considered to be a test with a higher specificity than the ELISA.

The correlation between the autoantibody titer of the existing test (IMESACUP) and the autoantibody titer detected by the A-cube was *p* = 0.0001 and r = 0.934, suggesting a good correlation (Figure 2C).

### 3.6. U1RNP and SNRNP70 

Of the 15 ELISA-positive cases, 12 were positive for SNRNP70, SNRPA, or SNRPC by the A-cube (Table 2). Out of the 82 ELISA-negative cases, all 82 were consistent with the A-cube (Table 2). We performed additional double immunodiffusion assays on the discordant cases and found that all three cases were negative, so they were considered to be false positive ELISA results. Therefore, the anti-U1RNP antibody was considered to be a highly specific test compared to ThermoFisher.

### 3.7. Anti-Jo-1 Antibody MESACUP-3

The anti-Jo-1 antibody MESACUP-3 was positive in two of the two cases confirmed by ELISA (MESACUP-3) (Table 2). Further, 34 of the 34 ELISA-negative cases were consistent with the 34 negative cases of the A-cube (Table 2).

### 3.8. Anti-TIF1γ Antibody

Of the nine patients that tested positive for the anti-TIF1γ antibody using ELISA (MESACUP) (Table 2), nine were A-cube-positive. In total, 10 out of 10 patients tested negative for ELISA, which was consistent with the 10 cases that tested negative for the A-cube (Table 2).

### 3.9. Anti-Mi-2 Antibody

One case of anti-Mi-2 antibody positivity tested using ELISA (MESACUP) was also positive for the A-cube (Table 2). Fifteen cases of negative ELISA results were consistent with fifteen cases of negative A-cube results (Table 2).

### 3.10. Anti-PL-7 Antibody

Four of the four patients who tested positive for the anti-PL-7 antibody using the A-cube tested positive using ELISA (Table 2).

### 3.11. Anti-PL-12 Antibody

ELISA was positive in one out of four patients who tested positive for the anti-PL-12 antibody using the A-cube (Table 2).

### 3.12. Anti-EJ Antibody

One patient with positive the anti-EJ antibody in the A-cube tested positive using ELISA (Table 2). 

### 3.13. Comparison with the Immunoprecipitation Method

Through the immunoprecipitation method, the anti-Th/To antibody, anti-U3RNP antibody, anti-NOR90 antibody, anti-PM-Scl antibody, anti-topoisomerase I antibody, anti-RNA polymerase I antibody, anti-RNA polymerase II antibody, anti-RNA polymerase III antibody, anti-Ku antibody, anti-Jo-1 antibody, anti-PL-7 antibody, anti-PL-12 antibody, anti-EJ antibody, anti-OJ antibody, anti-KS antibody, anti-SRP antibody, anti-SAE antibody, anti-TIF1α antibody, anti-TIF1β antibody, anti-TIF1γ antibody, anti-MXP2 antibody, and anti-Mi-2 antibody were detected in the samples from the cases and compared with the A-cube. The A-cube was able to detect the antibodies detected via immunoprecipitation in 60 of 61 cases, except for the anti-TIF1β antibody.

### 3.14. Cases in Which the Diagnosis Was Reconsidered by Using A-Cube

**Figure 3:**
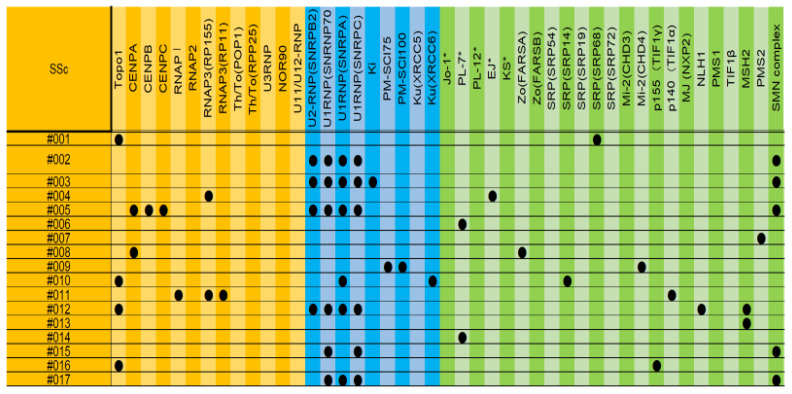
The results of cases in which no SSc-specific autoantibodies or DM-specific autoantibodies were detected in SSc. Autoantibodies marked with * are autoantibodies that are measured collectively in conventional tests. Autoantibodies in the orange area are autoantibodies that are more likely to be detected in SSc, autoantibodies in the green area are autoantibodies that are more likely to be detected in DM, and autoantibodies in the blue area are autoantibodies that are more likely to be detected in both or in overlap syndrome.

Figure 3 summarizes the results of the cases in which no SSc-specific autoantibodies or DM-specific autoantibodies were detected in SSc. 

When we reconsidered the diagnoses of these cases, we found that they had DM-specific skin rashes and findings suggestive of muscle weakness. In addition, the diagnosis of SSc only met the VEDOSS criteria [24] and did not meet any other diagnostic criteria. Therefore, these cases were followed up as SSc or SSc + DM, but it was considered appropriate to treat them as DM. This suggests that some of the cases diagnosed using the VEDOSS criteria may have actually been DM or PM.

As for the cases in which both SSc- and DM-specific antibodies were detected, upon re-examination, we found that there were five cases (#6, #9, #10, #14, #15, and #16) that were originally followed up as SSc + polymyositis (DM), and these cases were considered to be consistent with SSc + DM. Although #1–5, #11, and #18 were followed up as SSc, it was considered appropriate to treat them as SSc + DM.

**Figure 4:**
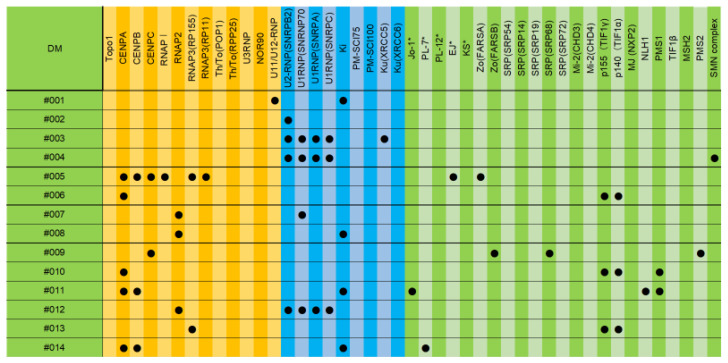
The results of cases in DM in which either DM-specific autoantibodies were not detected or SSc-specific autoantibodies were detected. Autoantibodies marked with * are autoantibodies that are measured collectively in conventional tests. Autoantibodies in the orange area are autoantibodies that are more likely to be detected in SSc, autoantibodies in the green area are autoantibodies that are more likely to be detected in DM, and autoantibodies in the blue area are autoantibodies that are more likely to be detected in both or in overlap syndrome.

Figure 4 summarizes the results of the cases of DM in which either DM-specific autoantibodies were not detected or SSc-specific autoantibodies were detected.

As a result of the re-examination of cases #1 and #12, the VEDOSS diagnostic criteria for SSc were met, and it was considered appropriate to treat them as SSc rather than DM [24]. The remaining cases were considered to be DM + SSc.

## 4. Discussion

The above results suggest that the A-cube is comparable with existing tests or has a high sensitivity or specificity. In addition, the A-cube was able to detect anti-OJ antibodies that could not be detected using conventional tests. 

Conventional tests have previously used *E. coli* for protein synthesis, but the number of proteins that can be synthesized is limited, as the prokaryotic organism has a different translation reaction pattern than humans [25,26,27]. 

The cell-free protein synthesis method maintains the same speed and accuracy of peptide synthesis as that in living cells, and because it does not use living organisms, it is not subject to physiological constraints and is expected to dramatically expand the range of synthesizable molecular species [28,29]. The use of the wheat cell-free protein synthesis method may have enabled the detection of anti-OJ antibodies, because proteins that could not be synthesized using conventional methods could be synthesized. In addition, the A-cube retained the higher-order structure of the protein by spotting a solution containing the undenatured antigen protein on the substrate and freezing it. The preservation of the higher-order structure of the enzyme complex containing IARS, which is considered to be a major anti-OJ antibody antigen, may have led to the recognition of anti-OJ antibodies [16,17].

Antibodies specific for SSc, such as anti-topoisomerase I, anti-centromere, and anti-RNAP3, are rarely detected when one of them is detected [30]. The clinical picture of systemic scleroderma is similar to that of SSc, even when multiple antibodies are present. In addition, the clinical picture of SSc strongly reflects the clinical picture of SSc-specific antibodies and is not affected by other antibodies, even if multiple antibodies are present [31]. In contrast, anti-centromere antibodies and anti-topoisomerase I antibodies are said to be co-positive at a rate of 0.05–5.6% [32,33,34,35,36,37]. According to an article that investigated cases of co-positivity between anti-topoisomerase I and anti-centromere antibodies, the rate of co-positivity was lower than the rate of co-positivity when anti-topoisomerase I and anti-centromere antibodies were assumed to exist independently without affecting each other. It is also known that, in co-positive cases, there are strong clinical findings of vascular and other visceral effects [33]. In addition, studies in New Zealand have suggested that antibody profiles differ between countries, such as Denmark, the US, Sweden, Canada, France, the UK, and Italy [38,39,40,41,42,43]. It is possible that a comprehensive analysis of these autoantibodies using the A-cube may clarify the characteristics of the antibody profile of SSc, which has been unknown until now. Moreover, a comprehensive analysis using the A-cube may enable a more accurate diagnosis of SSc or DM in cases that meet only the early diagnosis criteria, which are difficult to diagnose with existing tests.

In the treatment of SSc, skin sclerosis and abnormalities in capillaroscopy are important for diagnosis [44,45,46,47], but this judgment requires training and may be difficult to perform without SSc specialists. In particular, when CENPB, Topo1, and RNAP3 are not detected, even specialists may have difficulty in making this diagnosis, as we experienced in our case. Our hospital has a scleroderma center, is one of the leading hospitals in Japan for the treatment of SSc, and plays a central role in the treatment of SSc in Japan.

The A-cube is useful for SSc specialists, as well as SSc non-specialists, and may become a useful diagnostic tool for both types of clinicians.

In addition, it is noteworthy that there were cases in which the possibility of DM was suggested by a comprehensive analysis. Although it is recommended in current medical practice to perform only the necessary tests for a differential diagnosis, the results of this study suggest the usefulness of a comprehensive analysis in SSc treatment.

On the other hand, there were some cases of DM that were diagnosed as SSc or DM + SSc by a comprehensive measurement.

This suggests that SSc and DM are both difficult to differentiate from each other, and that a comprehensive analysis may facilitate differentiation between them.

As for the differentiation between SSc and DM, SSc-ILD is known to be more difficult to treat than other CTD-ILD, especially in pulmonary treatment [48], and the treatment strategies are different. Therefore, it is important to diagnose SSc and DM accurately.

In conclusion, the quality of the A-cube was assured and the usefulness of a comprehensive analysis was demonstrated by this study, thereby showing that the A-cube can contribute to the clinical treatment of SSc and DM.

## Figures and Tables

**Table 1:** Characteristics of systemic sclerosis, dermatomyositis, and healthy individuals participating in the study. SD: standard deviation. The age of the control group was 46.88 ± 16.95 and the male:female ratio of the control group was 46:44.

SSc					DM				
	Mean	SD	Physical Appearance (+:−)			Mean	SD		
Age (Year)	59.3	13.97	Sex	16:84	Age (Year)	58.92	14.82	Sex (+:−)	17:33
Duration (year)	10.89	9.78	diffuse cutaneous systemic sclerosis:limited cutaneous systemic sclerosis	70:29	Duration (year)	7.566	8.872		
Modified Rodnan skin score (mRSS)	9.06	9.33	Sclerodactyly	46:11	%Vital capacity (%)	92.19	17.19	Treatment (+:−)	
Right ventricular systolic pressure (mmHg)	28.74	13.72	Nail fold bleeding	50:21	%Diffusing capacity of the lung carbon monoxide (%)	91.62	17.26	Prednisolone	33:17
%Vital capacity (%)	83.42	18.42	Pitting scar	20:34	Krebs von den Lungen-6 (KL-6) (U/mL)	478.6	393.8	Immuno-suppressant	21:29
%Diffusing capacity of the lung carbon monoxide (%)	77.54	22.34	ulcer	29:18	surfactant protein-D (SP-D) (ng/mL)	130.5	102.5		
Krebs von den Lungen-6 (KL-6) (U/mL)	641.2	544	Raynaud’s phenomenon	64:14	White blood cell (/μL)	6568	2524		
surfactant protein-D (SP-D) (ng/mL)	153.9	132.3	telangiectasia	20:29	C-reactive protein (mg/L)	0.8034	2.905		
White blood cell (/μL)	7583	4012	calcinosis	7:39	Erythrocyte sedimentation rate (mm/h)	28.17	24.01		
C-reactive protein (mg/L)	0.81	2.32	Contracture of phalanges	20:23	estimated glemerular filtration rate (mL/min/1.73 m^2^)	79.85	20.19		
Erythrocyte sedimentation rate (mm/h)	22.26	24.14	arthralgia	16:28	Aldolase (IU/L)	9.385	7.473		
estimated glemerular filtration rate (mL/min/1.73 m^2^)	78.73	22.84	limited range of motion	6:1	D-dimer (μg/mL)	2.337	3.157		
Aldolase (IU/L)	6.379	2.194	Interstitial lung disease	63:32	Plasmin-α2 plasmin inhibitor complex (μg/mL)	1.19	0.8595		
D-dimer (μg/mL)	1.594	2.466	Gastroesophageal Reflux Dis-ease	70:17	Brain natriuretic pepride (pg/mL)	41.03	50.82		
Plasmin-α2 plas min inhibitor complex (μg/mL)	0.96	0.67	muscle disorder	10:12	Platelet (10^4^/μL)	23.47	9.125		
Brain natriuretic pepride (pg/mL)	110.1	195.5	heart failure	10:48	Ferritin (μg/L)	131.7	235.8		
Platelet (10^4^/μL)	25.97	13.13	kidney disease	7:90	Hemoglobin (g/dL)	12.52	1.931		
Ferritin (μg/L)	63.12	82.28	liver disease	5:93	Creatine Kinase	536	2537		
Hemoglobin (g/dL)	12.02	1.68	thyroid disease	9:33					
			Antiphospholipid antibody syndrome	4:21					
			Antiphospholipid antibody syndrome antibody	7:77					
			Sjögren’s syndrome	16:20					
			Pulmonary arterial hypertension	13:60					
			Treatment (+:−)						
			Prednisolone	47:53					
			Immunosuppressant	32:68					

**Table 2:** The results for each antibody. SD: standard deviation.

Autoantibody	Antigen	Average	SD	Specificity in 82 Healthy Controls (%)	Positive Concordance Rate with Existing Tests (%)	Negative Concordance Rate with Existing Tests (%)
Anti CENPA antibody (CENP-A)	Centromere protein A (CENPA)	2.3	2.1	100		
Anti CENPB antibody (CENP-B)	Centromere protein B (CENPB)	0.8	1.2	100	100 (20/20)	99 (74/75)
Anti CENPC antibody (CENP-C)	Centromere protein B (CENPB)	0.2	0.3	100		
Anti Topo|antibody	Topoisomerase I (Topo |)	0.3	0.4	100	97 (30/31)	100 (67/67)
Anti RNAPⅢ(RP155) antibody	RNA polymerase III subunit A (RNAPⅢ(RP155))	0.4	0.7	100	93 (13/14)	100 (75/75)
Anti RNAPⅢ(RP11) antibody	RNA polymerase III subunit C (RNAPⅢ(RP11))	0.7	1.3	100		
Anti RNAP|(POLR1A) antibody	DNA-directed RNA polymerase I subunit RPA1 (RNAP1(POLR1A))	0.3	0.3	100		
Anti RNAPⅡ(POLR2A) antibody	RNA Polymerase II Subunit A (RNAPⅡ(POLR2A))	1.5	1.9	100		
Th/To (7-2RNP)	Th/To ribonucleoprotein (POP1)	0.6	0.7	100		
Th/To (7-2RNP)	Th/To ribonucleoprotein (RPP25)	1.1	1.4	100		
U3-RNP (fibrillarin)	Fibrillarin	1.2	1.0	100		
hUBF (NOR90)	Nucleolus-organizing region	0.7	1.5	98.8		
U11/U12-RNP	RNPC3	0.2	0.3	100		
SSSCA1	Sjogren syndrome/scleroderma autoantigen 1 (SSSCA1)	1.0	1.5	100		
U1RNP	SNRNP70	0.7	1.5	100	80 (12/15)	100 (82/82)
U1RNP	SNRPA	0.4	0.9	100		
U1RNP	SNRPC	0.2	0.3	100		
U2-RNP	SNRPB2	0.8	1.7	98.8		
Ku	XRCC5	0.3	0.4	100		
Ku	XRCC6	0.6	0.6	100		
PM-SCl100	EXOSC10	1.4	1.4	97.6		
PM-SCl75	EXOSC9	0.3	0.5	100		
Ki	PSME3	0.4	0.5	100		
Jo-1*	HARS	0.7	1.0	100	100 (2/2)	100 (34/34)
PL-7*	TARS	0.4	0.7	100	100 (4/4)	
PL-12*	AARS	0.2	0.3	100	100 (1/1)	
EJ*	GARS	0.6	0.7	100	100 (1/1)	
KS*	NARS	0.4	0.5	100		
OJ	IARS	0.3	0.5	100		
Zo	FARSA	1.0	1.2	100		
Zo	FARSB	1.0	1.2	100		
SRP	SRP54	0.5	0.8	100		
SRP	SRP14	0.6	1.7	98.8		
SRP	SRP19	0.6	0.6	100		
SRP	SRP68	1.4	1.2	100		
SRP	SRP72	0.9	0.9	100		
Mi-2	CHD3	0.3	0.4	100		
Mi-2	CHD4	0.4	1.0	100	100 (1/1)	100 (15/15)
p155 (TIF1γ)	TRIM33	0.6	0.7	100	100 (9/9)	100 (10/10)
p140 (TIF1α)	TRIM24	1.1	1.4	100		
TIF1β	TRIM28	0.4	0.5	100		
MJ (NXP2)	MORC3	0.6	0.6	100		
SMN	SMN1	1.8	2.1	100		

## Data Availability

The data that support the findings of this study are available on request from Ayumi Yoshizaki.
